# Dr. Beard on Herbert Spencer

**Published:** 1883-04

**Authors:** 


DR. BEARD ON HERBERT SPENCER.

The recent death of Dr. George M. Beard will lend additional interest to the little pamphlet by him just published by the Putnams. It is entitled "Herbert Spencer on American Nervousness—A Scientific Coincidence." The seventeen pages are mainly taken up with showing how precisely Mr. Spencer's views on American nervousness, while he was in this country, corresponded to Dr. Beard's own views as previously made known in his volume on that subject published in 1881. Dr. Beard was not so conceited or so unjust as to take for granted that Mr. Spencer had privately adopted his conclusions, but the similarity of the language used by both encouraged Dr. Beard to suspect that this similarity of thoughts and words was merely an interesting coincidence. Anybody who understands the late Dr. Beard's character at all, knows that he was particularly honest, not only in his professional dealings, but in his announcement of his doctrines, beliefs and theories. He was many sided. The facts of his mind were numerous and bright. An unfortunate physical infirmity rendered it impossible that he should be able to be as good a practising physician as he was a writer on nervous disease and psychological disease. His little book now speaks with the solemnity and authority of a voice that has passed beyond the confines of human existence. It is to be hoped that his sudden and early death will not deprive the world of that "deep explanation," which he here confesses he had arrived at, whereby an elucidation is given of such inconsistent phenomena, as the co-existence of a chronic brain exhaustion and the highest and hardest brainwork through a long lifetime. Dr. Beard hints that he has already prepared an answer to this very interesting query. If so, it is to be hoped that it will be found among his posthumous papers. The question itself is entirely new and unheard of to most persons, and it cannot but be believed that so trained, so electric and so ingenious a mind as Dr. Beard's has much that is both startling and satisfactory to say in response.

